# Nonunion after medial malleolar osteotomy in total ankle arthroplasties for severe varus deformity: a report of 3 cases

**DOI:** 10.1093/jscr/rjae358

**Published:** 2024-05-30

**Authors:** Gensuke Okamura, Makoto Hirao, Takaaki Noguchi, Yuki Etani, Kosuke Ebina, Taihei Miura, Hideki Tsuboi, Atsushi Goshima, Seiji Okada, Jun Hashimoto

**Affiliations:** Department of Orthopaedic Surgery, National Hospital Organization, Osaka Minami Medical Center, Osaka 586-8621, Japan; Department of Orthopaedic Surgery, National Hospital Organization, Osaka Minami Medical Center, Osaka 586-8621, Japan; Department of Orthopaedic Surgery, Osaka University Graduate School of Medicine, Osaka 565-0871, Japan; Department of Orthopaedic Surgery, Osaka University Graduate School of Medicine, Osaka 565-0871, Japan; Department of Orthopaedic Surgery, Osaka University Graduate School of Medicine, Osaka 565-0871, Japan; Department of Orthopaedic Surgery, Osaka University Graduate School of Medicine, Osaka 565-0871, Japan; Department of Orthopaedic Surgery, Osaka Rosai Hospital, Osaka 591-8025, Japan; Department of Orthopaedic Surgery, Osaka Rosai Hospital, Osaka 591-8025, Japan; Department of Orthopaedic Surgery, Osaka University Graduate School of Medicine, Osaka 565-0871, Japan; Department of Orthopaedic Surgery, National Hospital Organization, Osaka Minami Medical Center, Osaka 586-8621, Japan

**Keywords:** total ankle arthroplasty, severe varus deformity, medial malleolar osteotomy, internal fixation, valgus tibial osteotomy

## Abstract

Of the three ankles after total ankle arthroplasty (TAA) with medial malleolar osteotomy for severe varus deformity (talar varus tilt >10°), two failed in varus migration of the tibial component. In these two cases, tibial osteotomy was performed with varus alignment of 5°and 2°, and with medially shifted placement of tibial component, while one ankle showed no migratoin of prostheses after 5 years, even with nonunion. In this case, tibial osteotomy was performed with a valgus alignment of 4°. Internal fixation after medial malleolar osteotomy should be done for severe varus cases. Medially shifted placement of tibial component should be avoided. Fortunately, the failure did not occur in a case of valgus of the distal tibia. Valgus tibial osteotomy might help to reduce the collision of the talus against the medial malleolus.

## Introduction

Total ankle arthroplasty (TAA) is performed to treat destruction of the ankle joint and deformities induced by osteoarthritis (OA) or inflammatory arthritis, including rheumatoid arthritis (RA). Severe varus deformity (talar varus tilt >10°) has generally been suggested to be a contraindication to TAA [1]. To overcome this problem, medial malleolar osteotomy was established by Doets et al [2]. Medial malleolar osteotomy can produce good outcomes after TAA for varus ankle deformity [3], but at the same time, it has also been reported that cases of TAA for varus deformity necessitating medial malleolar osteotomy have a higher failure rate than TAA for cases of regular deformity. As such, fixation with a stable prosthesis in good alignment is important [4]. Issues could arise with respect to dysfunction of the anterior talo-fibular ligament (ATFL) with such a severe varus deformity, especially in the case of an old ankle sprain. Although the threshold for the grade of ATFL dysfunction is unknown, if ATFL function is impaired beyond a certain level, it is expected that braking the talus toward the lateral direction could become completely ineffective, and, subsequently, the talus would repeatedly collide with the medial malleolus during gait and weight-bearing, resulting in nonunion at the medial malleolar osteotomy. Indeed, in the present study, two failed cases with medial migration of the tibial component with nonunion after medial malleolar osteotomy, and one successful case (no loosening) even with nonunion after medial malleolar osteotomy for severe varus ankle deformity are presented, and their commonalities and differences are evaluated.

## Report of the cases

Three ankles had undergone treatment with a mobile-bearing ankle prosthesis (FINE mobile-bearing prosthesis; Teijin-Nakashima Medical Co., Okayama, Japan) [3] for the treatment of end-stage ankle deformity/destruction. The demographic characteristics of the patients and their radiographical outcomes/findings are shown in Table 1. This research was performed in compliance with the Declaration of Helsinki and was approved by the institutional review boards of the institutions affiliated with the authors. Written, informed consent was obtained from all patients.

**Table 1:** Clinical characteristics and radiographical outcomes/findings of the three cases

Variable	Case 1	Case 2	Case 3
Age (y)	72	76	75
Sex	Male	Female	Female
Follow-up period (months)	49	42	60
Preoperative diagnosis	Ankle OA	Ankle OA + RA	Ankle OA
Duration of disease (y)	-	25	-
Past surgical history	Lt. UKA	-	-
Steinbrocker’s stage/functional class	-	III/ I	-
Biologics	-	-	-
MTX dose (mg/week)	-	6	-
Prednisolone dose (mg/day)	-	0	-
Preoperative talar tilt (degrees) Opening of the lateral gutter	11 (varus) Yes	17 (varus) Yes	21 (varus) Yes
Surgical technique	TAA + medial malleolar osteotomy	TAA + medial malleolar osteotomy	TAA + medial malleolar osteotomy
Internal fixation at malleolar osteotomy Situation of bone union at the site of malleolar osteotomy after TAA Positioning of the tibial component at TAA (degrees) Closing of the lateral gutter after TAA Prosthetic migration/loosening	No Non 5 (varus) No Yes	No Non 2 (varus) No Yes	No Non 4 (valgus) Yes No

OA, osteoarthritis; RA, rheumatoid arthritis; Lt., left; UKA, unicompartmentall knee arthroplasty; MTX, methotrexate; TAA, total ankle arthroplasty

### Case 1

**Figure 1:**
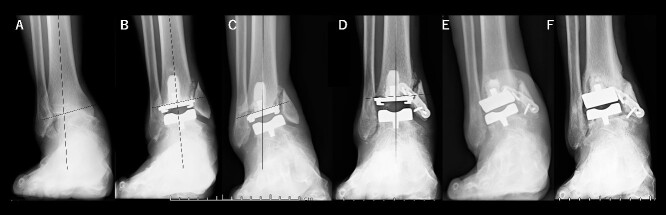
Radiographs of preoperative weight-bearing radiograph of the antero-posterior ankle joint in Case 1. (A) Preoperative. Talar tilt is 11° varus. Opening of the lateral gutter with os subfibulare is seen. (B) One month after surgery. Medial malleolar osteotomy without internal fixation, and tibial component placed in the 5° varus position. Opening of the lateral gutter still remains. (C) Five months after surgery. The tibial component shows varus migration, and nonunion at the medial malleolar osteotomy has occurred. Opening of the lateral gutter is exacerbated. (D) One month after revision surgery. The site of the medial malleolar osteotomy is fixed with a mini-plate, and varus positioning of the tibial component still remains (3° varus). The lateral gutter is closed. (E) Three months after revision surgery. Varus migration of the tibial component has occurred again, and bone union at the osteotomy is still not seen. (F) Four years after revision surgery. Bone union has been achieved, but both tibial and talar components show malposition. The lateral gutter is still closed.

A 72-year-old man with end-stage ankle OA and severe ankle joint pain and gait dysfunction had a JSSF ankle/hindfoot scale score of 42 points. Talar tilt was 11° varus (Fig. 1A). He underwent TAA with medial malleolar osteotomy without internal fixation, and the tibial component was placed in the 5° varus position (Fig. 1B). Within 6 months, the tibial component showed varus migration, and nonunion at the medial malleolar osteotomy occurred (Fig. 1C). Revision TAA with mini-plate fixation at the medial malleolus was then performed (Fig. 1D), but the tibial component was still placed in the varus position (3° varus); subsequently, varus migration of the tibial component occurred again (Fig. 1E). After that, vitamin D administration and low intensity pulsed ultrasound treatment were continued for more than 1 year, and bone union was achieved, but both the tibial and talar components showed malposition (Fig. 1F). At present, four years after revision TAA, he can walk for more than 1.5 hours and play golf.

### Case 2

**Figure 2:**
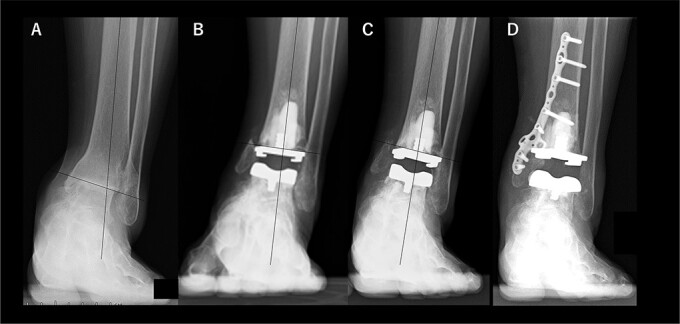
Radiographs of preoperative weight-bearing radiograph of the antero-posterior ankle joint in Case 2. (A) Preoperative. Talar tilt is 17° varus. Opening of the lateral gutter is seen. (B) One month after surgery. Medial malleolar osteotomy without internal fixation and the tibial component placed in the 2° varus position. Opening of the lateral gutter remains. (C) Three years after surgery. Varus migration of the tibial component has progressed after the patient missed the stairs (7° varus position of the component), and nonunion at the medial malleolar osteotomy has occurred. Opening of the lateral gutter has also remained. (D) Three months after revision surgery. Medial malleolar osteotomy site is fixed with a plate, and varus positioning of the tibial component still remains (3° varus). The lateral gutter is closed.

A 76-year-old woman with a 25-year history of RA [Clinical Disease Activity Index (DAS28-CRP score) 2.45: good control] had a JSSF ankle/hindfoot scale score of 42 points. She had been treated with 6 mg of methotrexate (MTX) per week. She complained of difficulty walking and ankle pain, resulting in an awkward gait. Talar tilt was 17° varus (Fig. 2A). She underwent TAA with medial malleolar osteotomy without internal fixation, and the tibial component was placed in the 2° varus position (Fig. 2B). Three years after TAA, she missed two or three steps while walking down the stairs. and developed anterior tibial pain. Radiography showed that varus migration of the tibial component had progressed (7° varus position; Fig. 2C), and she required revision TAA with internal fixation using a plate and allograft bone transplantation (Fig. 2D).

### Case 3

**Figure 3:**
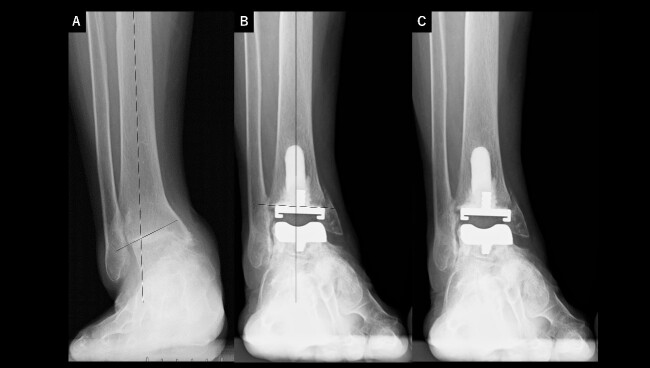
Radiographs of preoperative weight-bearing radiograph of the antero-posterior ankle joint in Case 3. (A) Preoperative. Talar tilt is 21° varus. Opening of the lateral gutter is seen. (B) One month after surgery. Medial malleolar osteotomy without internal fixation and the tibial component placed in the 4° valgus position, unlike the other two cases. The lateral gutter is closed. (C) Five years after surgery. There is no evidence of prosthetic loosening, even though incomplete bone union at the osteotomy site is seen. The lateral gutter is still closed.

A 75-year-old woman with end-stage ankle OA and severe ankle joint pain and gait dysfunction had a JSSF ankle/hindfoot scale score of 42 points. Talar tilt showed 21° varus (Fig. 3A). She underwent TAA with medial malleolar osteotomy without internal fixation, and the tibial component was placed in the 4° valgus position, unlike the other two cases (Fig. 3B). At present, 5 years after TAA, she can walk for more than 2 hours and feels no pain around the ankle. There is no evidence of prosthesis loosening even though incomplete bone union at the osteotomy site is evident (Fig. 3C).

## Discussion

In this study, nonunion after medial malleolar osteotomy at TAAs for severe varus ankle deformity was evaluated in three cases. As commonalities, all three cases showed nonunion at the medial malleolar osteotomy site, and no internal fixation had been applied, suggesting the necessity of internal fixation in severe varus deformity cases to achieve reliable bone union. On the other hand, as a difference, the tibial component was placed in the varus position in each implant migration case (5° and 2° varus), but it was in the valgus position in the successful case (4° valgus) even with nonunion, subsequently closing of lateral gutter was achieved, suggesting that valgus osteotomy of the distal plafond of the tibia could contribute to tilting the talus outward (lateral malleolus), not inward (medial malleolus), decreasing the collision of the talus against the medial malleolus, and subsequently bone union could be more easily achieved with the newly formed, stable ankle joint. Interestingly, it is known that the chimpanzee ankle joint does not have a lateral ligament such as the ATFL, because of the morphology of the chimpanzee ankle joint; the distal plafond of the tibia clearly exhibits a valgus shape [5, 6]. As a result of chimpanzees’ lifestyle, the practical range of motion of the chimpanzee ankle tends to dorsiflexion for climbing trees, not for upright walking, and thus the chimpanzee ankle exhibits a structurally stable shape [6]. Because of this stable shape with a valgus plafond, there is no need for a structure such as the ATFL to exist. These concepts are similar to the theory of distal tibial oblique osteotomy (DTOO) [7]. Oblique osteotomy results in lateralization of the medial malleolus and talus by making the distal plafond valgus, with subsequent stability of the talus provided by the lateral malleolus. There is also no need for the ATFL in the newly formed ankle joint after DTOO. We have had previous experience with several cases involving patients who underwent additive ATFL reconstruction for severe varus deformity (data not shown), but in these cases there occurred a situation under which implantation of the internal brace for the reconstruction was difficult because of osteoporosis due to long-term stress concentration toward the medial side. It was then considered that if the tibial plafond were to be shaped into valgus, the need to reconstruct the ATFL could be reduced. Although a distal tibial osteotomy is meant to be perpendicular to the longitudinal axis of the tibia, there is no definitive rule for tibial osteotomy; thus, it is plausible to consider that valgus osteotomy of the distal tibia during TAA could be applied in cases of severe varus deformity cases. In this way, it is similar to tibial osteotomy during total knee arthroplasty (TKA). In tibial osteotomy during TKA, the concept of both conventional mechanical alignment and kinematic alignment coexists [8, 9]. In the successful case even with nonunion in the present study, valgus osteotomy of the distal tibia caused no prosthetic migration 5 years after TAA (Case 3), and there was no pain at the site of medial malleolar osteotomy, even though incomplete bone union was seen. Thus, it is feasible and safe to perform valgus osteotomy of the distal tibia in very severe varus cases, but internal fixation at the site of medial malleolar osteotomy is required. Further observation and discussion with more increased number of cases and a longer follow-up period are required. Knee alignment, especially varus knee alignment, also should be put into consideration in the future. In addition, the precise degrees of valgus osteotomy should be further discussed in the future. At the same time, positioning of tibial component was not also ideal in the two failed cases: medially shifted placement of tibial component. Patient specific instrument also should be discussed to be utilized in such severe varus deformity cases to avoid the malpositioning (medially shifted placement) of the tibial prostheses.

In conclusion, based on this small number of cases, internal fixation at the site of medial malleolar osteotomy should be done for severe varus cases. Valgus osteotomy of the distal tibia might contribute to preventing the medial concentration of the loading pressure.

## Conflict of interest statement

None declared.

## Funding

This work did not receive any grants from funding agencies in the public, commercial or not-for-profit sectors.

## Ethical statement

This research was performed in compliance with the Declaration of Helsinki. Ethical approval for this study was obtained from Institutional Review Board of National Hospital Organization, Osaka Minami Medical Center (approval number: R4-28) and Osaka University Hospital (approval number: 14219). Written, informed consent was obtained from all patients.

## Financial benefits to the authors

None of the authors have any commercial or financial involvements in connection with this study.
